# Internal treatment in traditional Chinese medicine for patients with COVID-19: A protocol for systematic review and meta-analysis

**DOI:** 10.1097/MD.0000000000023739

**Published:** 2020-12-18

**Authors:** Mingxu Zhang, Ju Huang, Xinhui Wu, Lizeyu Lv, Anming Huang, Siquan Zhu

**Affiliations:** a Eye School of Chengdu University of Traditional Chinese Medicine; b College of Acupuncture and Tuina, Chengdu University of Traditional Chinese Medicine; c Hospital of Chengdu University of Traditional Chinese Medicine, No. 39 Shi-er-qiao Road, Chengdu, Sichuan Province, PR; d Department of Ophthalmology, Beijing Anzhen Hospital, Capital Medical University, Beijing, China.

**Keywords:** COVID-19, internal treatment, systematic review, traditional Chinese medicine

## Abstract

**Background:**

The safety and effectiveness of Internal Treatment in Traditional Chinese Medicine (TCM) on Corona Virus Disease 2019 (COVID-19) is the main subject of this protocol for systematic review and meta-analysis.

**Methods:**

The following online databases will be searched from inception to April 2020: Cochrane Central Register of Controlled Trials, PubMed, Web of Science, EMBASE, China National Knowledge Infrastructure, Traditional Chinese Medicine, Chinese Biomedical Literature Database, Wan-Fang Database, and Chinese Scientific Journal Database. All published randomized controlled trials in English or Chinese related to Internal Treatment in Traditional Chinese Medicine for COVID-19 will be included. Primary outcomes are time of disappearance of main symptoms and serum cytokine levels. Secondary outcomes is Accompanying symptoms disappear rate, negative COVID-19 results rate on 2 consecutive occasions CT image improvement, average hospitalization time, occurrence rate of common type to severe form, clinical cure rate, and mortality. Two reviewers will conduct the study selection, data extraction, and assessment independently. The assessment of risk of bias and data synthesis will be conducted with Review Manager Software V.5.2.

**Results:**

The results will provide a high-quality synthesis of current evidence for researchers in this subject area.

**Conclusion:**

The conclusion of our study will provide evidence to judge whether the internal treatment in traditional Chinese medicine is an effective intervention for COVID-19 patients.

**PROSPERO registration number:**

CRD42020180178.

## 1 Introduction

With the sudden incidence and rapid transmission of COVID-19, the pandemic has affected more than 38 million patients and caused more than 1 million deaths globally as of October 2020 according to the report of World Health Organization (WHO).^[1]^ The symptoms of COVID-19 are various and atypical, but the most common symptoms are cough, headache, fever, and diarrhea, which are difficult to distinguish from respiratory infections caused by other pathogens.^[2,3]^ SARS-CoV-2, a novel coronavirus, has been confirmed to be the pathogen of COVID-19, and it targets the Angiotensin-Converting Enzyme 2 (ACE2) to invade host cells.^[4]^ Due to the lack of effective treatment and highly contagious, COVID-19 resulted in an unexpected chaos globally, and threatened peoples current trust in public health administration. For the present diagnosis rules of COVID-19, the criteria of confirmation relies on the positive results of PCR technique, but its sensitivity and specificity need further improvement.^[5]^ But the computed tomography can also provide valuable significance for clinicians to diagnose early phase of COVID-19.^[6]^

The treatment on COVID-19 contains herbs, chemicals and biologics, but the proven effective therapy is still absent. The Remdesivir, a kind of adenosine nucleoside triphosphate analogue antiviral agent which can reduce viral RNA reproduction by interfering with viral RNA-dependent RNA polymerase, exhibited antiviral effects on SRAS-CoV-2 in vivo.^[7]^ But the first randomized clinical trial for the Remdesivir on COVID-19 failed to provide significant result and positive suggestion for clinical practitioners globally.^[8]^ The Lopinavir and Ritonavir are usually used to treat the infection of human immunodeficiency virus, and were thought to be the candidate therapy for SARS-CoV-2. But a clinical trial performed Cao showed that the lopinavir-ritonavir treatment failed to improve clinical symptom and serum assays of patients with COVID-19.^[9]^ The cytokine storm and inflammatory reaction are serious threat for severe patients with COVID-19. The randomized clinical trial performed by Sofia exhibited that high-dose methylprednisolone can effectively accelerate respiratory recovery and reduce hospital mortality in patients with COVID-19 associated cytokine storm syndrome.^[10]^

Traditional Chinese Medicine (TCM) are widely used in China to treat patients with COVID-19, and amounts of clinical trials have been performed by TCM-practitioners.^[11]^ The internal treatment in TCM include single drug and drug compounds via oral/injection administration, and is the most common therapy in TCM. According to the clinical features of COVID-19, specialists of TCM accused the symptoms of COVID-19 to pathogenic dampness.^[12,13]^ Chens human experimentation shows that the active components of Lianhuaqingwen capsule (a Chinese patent medicine contained multiple components of herbs) can effectively target ACE2 which means a potential preventive and therapeutic effects on COVID-19 infections.^[14]^ Some bioinformatics research were also suggested that many TCM compounds or patent medicines contained multiple active components which could target COVID-19 related functional proteins.^[15,16]^ In a large-scale randomized clinical trial, 2 Chinese patent medicines (Huoxiangzhengqi oral liquid and Jinhaojiere granules) could exert preventive effects against COVID-19 in community populations.^[17]^ Such studies provide us with the potentials of Internal treatment in TCM to restrict the pandemic of COVID-19.

Due to the latent period and asymptomatic carriers, SARS-CoV-2 transmits rapidly among people and has caused bulks of secondary disasters globally.^[18,19]^ And the frequent mutations of SARS-CoV-2 not only increase the difficulties in the control of pandemic, but also set lots of obstacles in the research and development of specific agents and vaccines.^[20,21]^ With the considerable amounts of COVID-19 patients involved in the TCM prescription treatment in China, it is urgent for an unbiased evaluation on the therapeutic effects and efficacy of internal treatment in TCM.^[22]^ On the other hand, the safety of internal treatment in TCM is also important for the general application of TCM due to the possible drug-related liver injury.^[23]^ There are not effective TCM-treatments verified to cure COVID-19. It is necessary to perform a systematic review to provide a confirmed result from limited evidence from current researches.

## 2 Methods

### 2.1 Study registration

The systematic review protocol has been registered in PROSPERO. The registration number: CRD42020180178. The consent of this protocol report is based on the Preferred Reporting Items for Systematic Reviews and Meta-Analyses Protocols (PRISMAP) statement guidelines.^[24]^

### 2.2 Inclusion criteria for study selection

#### 2.2.1 Type of study

We will include articles related to Traditional Chinese medicine therapy of patients for COVID-19. Due to language restrictions, we will search for articles in English and Chinese in order to get a more objective and true evaluation, all articles included are randomized controlled trial (RCT) type articles.

#### 2.2.2 Type of participant

All patients for COVID-19 will be included regardless of sex, age, race, education, and economic status. Pregnant women, postoperative infections, psychopaths, patients with severe cardiovascular and liver and kidney diseases will not be included.

#### 2.2.3 Type of intervention

Traditional Chinese medicine therapy including Chinese herbal medicine and its prescription, while other traditional Chinese therapies such as tuina, moxibustion, cupping and acupuncture will be excluded. We will compare the following interventions: treatments other than Traditional Chinese medicine (e.g., usual or standard care, placebo, wait-list controls).

#### 2.2.4 Type of outcome measure

Primary outcomes: Time of disappearance of main symptoms (including fever, asthenia, cough disappearance rate, and temperature recovery time), and serum cytokine levels. Secondary outcomes: Accompanying symptoms (such as myalgia, expectoration, stuffiness, runny nose, pharyngalgia, anhelation, chest distress, dyspnea, crackles, headache, nausea, vomiting, anorexia, diarrhea) disappear rate, negative COVID-19 results rate on 2 consecutive occasions (not on the same day), CT image improvement, average hospitalization time, occurrence rate of common type to severe form, clinical cure rate, and mortality.

### 2.3 Data sources

The following electronic databases will be searched from inception to April 2020: the Cochrane Central Register of Controlled Trials (CENTRAL), PubMed, EMBASE, Web of Science, China National Knowledge Infrastructure, Chinese Biomedical Literature Database, and Wan-Fang Database. About other sources, we also plan to manually search for the unpublished conference articles and the bibliography of established publications.

### 2.4 Search strategy

The search terms on PubMed are as follows: Traditional Chinese medicine (e.g., “Chinese medicine” or “herbs” or “TCM”); COVID-19 (e.g., “Corona Virus Disease 2019” or “Corona Virus”); randomized controlled trial (e.g., “randomized” or “randomly” or “clinical trial”). Combinations of Medical Subject Headings (MeSH) and text words will be used. The same search term is used in electronic databases in China. These search terms are shown in Table 1.

**Table 1:** Search strategy for the PubMed database.

Number	Search items
1	Chinese medicine
2	Herbs
3	Traditional Chinese medicine
4	TCM
5	1 or 2–4
6	COVID-19
7	Corona Virus Disease 2019
8	Corona Virus
9	6 or 7–8
10	Randomized controlled trial
11	Randomized
12	Randomly
13	Clinical trial
14	10 or 11–13
15	5 and 9 and 14

### 2.5 Data collection and analysis

#### 2.5.1 Selection of studies

**Figure 1:**
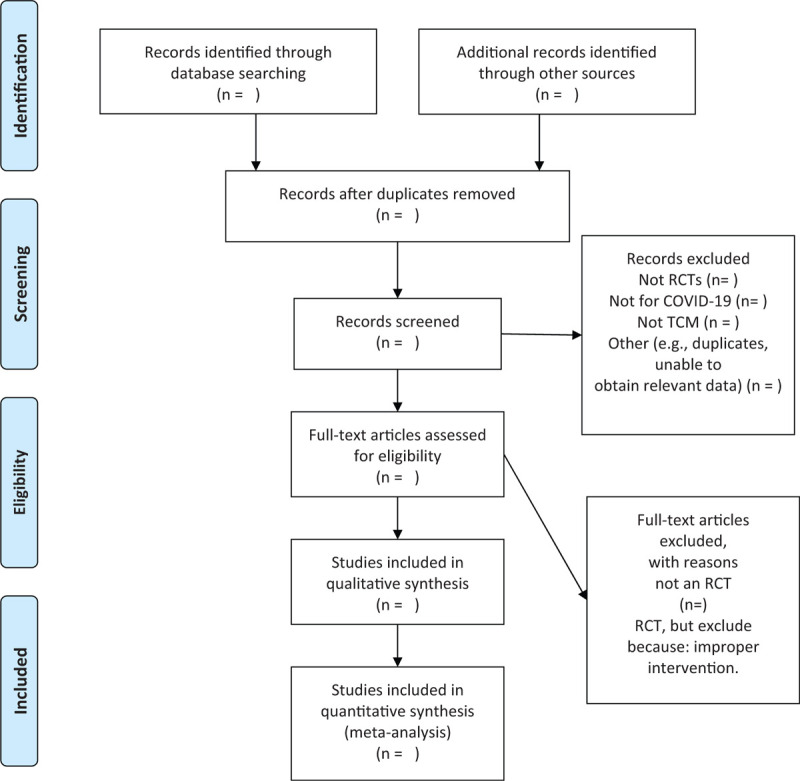
Flow chart of the study.

We chose the PRISMA flow chart to show the process of selecting literature for the entire study (Fig. 1). Before searching the literature, all reviewers will discuss and determine the screening criteria. After the screening requirements are clearly defined, the 2 reviewers will independently review and screen the literature. They screened the titles and abstracts of the literature, in order to get qualified studies, and then excluded some duplicate studies or studies with incomplete information. We will also try to obtain the full text, and the obtained literature will be managed by using EndNote software, V.X8 (United States). In case of disagreement between the 2 reviewers, discussions will be held with the third author for arbitration.

#### 2.5.2 Data extraction and management

The authors will strictly follow the inclusion criteria and select RCT articles related to the topic. Through the analysis of the article, we know participants characteristics (height, weight, sex), interventions, outcomes, the study characteristics (press, nationality, journals, research design), adverse reactions, etc. If there is any disagreement between the 2 authors in the literature data extraction, a third article participant will discuss the decision together. If there is a lack of data in the literature, we will contact the author or publisher as much as possible.

#### 2.5.3 Assessment of risk of bias in included studies

We will use the Cochrane collaborative tool to independently assess the risk of bias in the included studies. We will evaluate the following aspects of the article: sequence generation, assignment sequence hiding, blindness of participants and staff, outcome evaluators, incomplete result data, selective result reporting, and other sources of bias. The risk of bias is evaluated at 3 levels, namely, low risk, high risk, and ambiguity. If the information is vague, we will try to contact the author of the article.

#### 2.5.4 Measures of treatment effect

In this protocol, we will use 95% confidence interval (CI) risk ratio (RR) to rigorously analyze the dichotomous data. And for the continuous data, mean difference (MD) or standard MD (SMD) is used to measure the efficacy of 95% CI.

#### 2.5.5 Unit of analysis issues

We will include data from parallel group design studies for meta-analysis. In these trials, we will collect and analyze individual measurements of each outcome for each participant.

#### 2.5.6 Management of missing data

We will try our best to ensure the integrity of the data. If the included RCT data is not complete, we will try every means to contact the corresponding author of the article, including sending emails or making a phone call. If the corresponding author cannot be contacted, we will remove the experiment with incomplete data. After data integrity is assured, intention analysis therapy and sensitivity analysis will be performed.

#### 2.5.7 Assessment of heterogeneity

For the detection of heterogeneity, the I^2^ test will be used to detect the heterogeneity among trials. When the *I*^2^ test value is <50% and *P* value >1, we think there is no heterogeneity between these trials, and when the *I*^2^ test value is >50% and the *P* value is <1, there is significant heterogeneity between these included trials. If significant differences are detected, we will analyze the possible causes of heterogeneity, and then we can use the random effects model.

#### 2.5.8 Assessment of reporting biases

In this analysis, once >10 trials are included, funnel plots could be used to test for reporting bias.

#### 2.5.9 Data synthesis

We will use Review Manager Software (RevMan) V.5.3 (Copenhagen, Denmark) for data analysis and quantitative data synthesis. If there is no finding of statistical heterogeneity, the fixed-effect model is used for data synthesis. If there is significant statistical heterogeneity, we will use the random effect model, and all participants will explore the possible causes from a clinical and methodological perspective and provide a descriptive or subgroup analysis.

#### 2.5.10 Subgroup analysis

Subgroup analysis will be performed to explain heterogeneity if possible. Factors such as different types of control interventions and different outcomes will be considered.

#### 2.5.11 Sensitivity analysis

Based on sample size, study design, heterogeneous quality, methodological quality, and statistical model, sensitivity analysis will be performed to exclude trials with quality defects and ensure the stability of the analysis results.

#### 2.5.12 Grading the quality of evidence

This paper will use the evidence quality rating method to evaluate the results obtained from this analysis. GRADE is generally applied to a large amount of evidence. It has 4 evaluation levels, namely, high, medium, low, and very low. GRADE was used to evaluate the bias, inconsistencies, discontinuities, and inaccuracies of test results. In the context of the system review, quality reflects our confidence in the effectiveness of assessment.^[25]^

#### 2.5.13 Ethical review and informed consent of patients

Ethics and dissemination: The content of this article does not involve moral approval or ethical review and will be presented in print or at relevant conferences.

## 3 Discussion

This review is divided into 4 parts: identification, literature inclusion, data extraction, and data synthesis. It will systematically review the RCT literature; this review will evaluate the effectiveness of Internal Treatment in Traditional Chinese medicine in treating COVID-19 convalescent patients. There are also limitations in our research and the language bias here is that we only search for Chinese and English documents. Our study may provide a basis for clinicians to choose replacement therapy for further study in the future.

## Author contributions

All authors have read and approved the final manuscript.

**Conceptualization:** Mingxu Zhang, Ju Huang.

**Data curation:** Mingxu Zhang, Ju Huang.

**Funding acquisition:** Siquan Zhu.

**Investigation:** Xinhui Wu.

**Methodology:** Lizeyu Lv.

**Project administration:** Lizeyu Lv, Siquan Zhu.

**Resources:** Ju Huang, Xinhui Wu, Siquan Zhu.

**Software:** Mingxu Zhang.

**Supervision:** Siquan Zhu.

**Visualization:** Xinhui Wu.

**Writing – original draft:** Mingxu Zhang, Ju Huang, Xinhui Wu.

**Writing – review & editing:** Mingxu Zhang, Ju Huang, Siquan Zhu.
